# Gender-transformative school-based sexual health intervention: study protocol for a randomized controlled trial

**DOI:** 10.1186/s13063-024-08191-w

**Published:** 2024-06-05

**Authors:** Loreto Leiva, Betzabé Torres-Cortés, Andrés Antivilo-Bruna, Gloria Zavala-Villalón

**Affiliations:** 1 Department of Psychology, University of Chile, Santiago, Chile; 2 Independent Researcher, Madrid, Spain

**Keywords:** Randomized controlled trial, Adolescents, Sexual health education, School-based sexual health intervention, Gender perspective

## Abstract

**Background:**

There is general agreement that sexuality is a central aspect of human development; it is key in sexual health and reproductive education during adolescence. However, in spite of the existing interventions on this topic and the evidence generated, the inclusion of a gender focus in sexuality is relatively recent and there is little evidence available, thus structured and corroborated interventions with a gender-transforming perspective in sexuality are required.

**Methods:**

We will use a mixed method experimental design with a parallel cluster-randomized trial (GRTs) that will evaluate the effectiveness of a comprehensive gender-transformative intervention of sexual education (ENFOCATE -Focus-on-), which will be complemented with qualitative studies to understand the implementation process. The participants will be 609 10^th^ and 11^th^-grade students. The randomization will be by grade, and the data will be collected at three moments (pre-intervention, post-intervention, and a 3-month follow-up).

**Discussion:**

Comprehensive, gender-focused, and culturally pertinent interventions in sexuality are needed for adolescents of countries with high, middle, and low incomes. These produce better results in terms of sexual health, and including a gender-transformative focus contributes to equity in health. Focus-on is unique since it uses a comprehensive gender-transformative intervention in sexual education that will allow putting into practice a program based both on international evidence and that which arises from the object population. It also uses a culturally-sensitive focus, since it is designed based on the characteristics of the object population; it will allow adapting some activities to the needs of the context in which it is developed.

**Trial registration:**

The study was prospectively registered on June 6, 2023, at ClinicalTrials.gov ID: NCT05896540. Protocol version number 1.0. May 22, 2023.

**Supplementary Information:**

The online version contains supplementary material available at 10.1186/s13063-024-08191-w.

## Background

Adolescence is a sensitive stage in sexual health. Sexual initiation occurs frequently in this stage, which may be associated with greater risks [1]. Adolescents generally face difficulty in accessing information about sexual health and education [2], which is worse for some groups and areas [3]. In Latin America, this difficulty interacts with other factors such as the ethnic group, school dropouts, and income level [4].

One way to approach this topic is to include preventive interventions of sexual education in schools, which is one of the strategies most used to deal with it [5]. This type of intervention may be classified into three large groups: (1) interventions that focus on abstinence, promoting the delay of initiation of sexual relations until marriage, and providing little or no information on methods to prevent pregnancy or sexually transmitted infections (STI) [6]; (2) interventions that focus on risk prevention, which focus specifically on biological aspects of risky behavior in sexuality [7]; and (3) interventions with a comprehensive perspective, which include a holistic vision of sexuality appropriate to the life cycle and whose objective is to equip children and adolescents according to their capacities, providing them with knowledge, abilities, attitudes and values that allow them to develop a positive view of their sexuality [8].

The comprehensive interventions incorporate a teaching process based on cognitive, emotional, physical, and social aspects of sexuality, along with promoting the development of respectful social and sexual relations. They allow considering how their choices affect their well-being and that of others and understanding and guaranteeing the protection of rights throughout their lives. They also provide information about abstinence, how to practice safe sex, and avoid pregnancies and STI [6].

The evidence indicates that the “only abstinence” focus has shown to be less effective than comprehensive sexual education programs, since the latter contribute to retarding the beginning of sexual relations and decrease the number of sexual partners, increase the use of condoms and contraceptives, and promote gender equity [9]. Comprehensive interventions provide precise and appropriate information for different ages, develop abilities, and provide support to cope with the thoughts, feelings, and experiences that accompany sexual maturity [10]. They incorporate biological, psycho-social, and value aspects such as adolescent pregnancy, HIV/STI, sexual diversity, intra-family violence, and masturbation [11]. They also include a perspective of human and gender rights, contributing to increase self-esteem, develop better attitudes towards gender, and strengthen decision-making and communication abilities [6, 11].

However, in spite of the scientific knowledge about the results of comprehensive programs of sexual education, interventions with this focus have been less studied than those of abstinence [12]. Also, not all studies include the gender focus in a consistent manner [13], thus there is a need for more evidence from those studies that do incorporate it [12]. There are also no recent high-quality studies on the elements of its implementation, either in low- and middle-income countries [14] or in the world in general [3].

Interventions in the health area, although they include a gender focus, have difficulty in approaching gender as a determinant of health [15], thus changes in gender roles, relations, and practices need to be included as part of the main purposes of these interventions. This would allow the development of gender-transforming interventions, which consider gender as a factor that affects health, and of actions to approach the components of gender that may damage health [16]; interventions in sexuality are especially relevant, due to the intimate relation between sexual health and gender [17].

Comprehensive gender-transforming interventions in sexual education are especially relevant in Latin America, since the culture is still conservative in this region, and gender norms and attitudes are considered determinants of adolescent sexual health [18]. They are also needed to revert interventions centered on heteronormativity that promote gender inequality [19], distortion in the understanding of sexual orientation, discrimination against peers with different sexual orientations [7], and the development of stereotypes [20].

This kind of approximation has been recommended for scientific research [21], due to the impact that gender may have on health and because it allows for amplifying the external validity of the results [22]. Its use in sexual health is also especially sensitive in adolescents, since the way gender is approached would affect the outcomes of intervention [7].

The challenges mentioned above generated the design of a comprehensive gender-transforming intervention in sexual health education oriented to the adolescent school population called Focus-on, and a mixed experimental design to evaluate its effectiveness and implementation. Focus-on will have a comprehensive gender-transformative perspective, with a participative and reflexive methodology, as well as self-care strategies from abstinence to the practice of safe sex. This intervention will respond to the specific needs of the adolescent population and will be culturally pertinent and situated.

### Objectives of the study

The main objective of this study is to evaluate the effectiveness of a preventive-comprehensive program of sexual education in schools directed to students, using a mixed experimental design [23].

Given that comprehensive programs of sexual health have more effective results in attitudes and knowledge, and that a program that incorporates a gender-transformative perspective contributes to equity in sexual health, we hypothesize that the intervention designed and implemented in the experimental group: (i) will increase their sexuality knowledge level; (ii) will increase their preventive sexual behavior (or intentions); (iii) will favor a gender-positive attitude; and (iv) will decrease internalizing and externalizing problems and attention in mental health. The secondary objective is to examine the implementation intervention process, that is, identify the strengths and obstacles in the real context in which the study intervention is performed, to understand the determinants and strategies of the success of the implementation.

## Methods

### Trial design

**Fig. 1:**
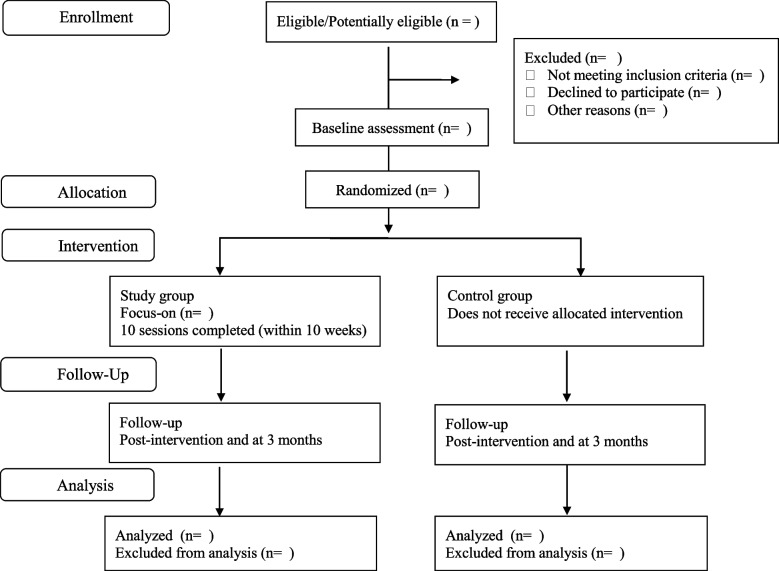
Flow chart of the trial profile of the study

We propose a mixed method experimental design [23] with a parallel cluster-randomized trial (GRTs) that will evaluate the efficiency of intervention and implementation of a comprehensive gender-transformative sexual education, along with qualitative studies to understand the implementation process. The results of the intervention will be evaluated after it finishes, and in a 3-month follow-up. Each participant will be sent a link to the questionnaire (web-based survey). Follow-up contact will be done with WhatsApp or telephone calls. The protocol of the study is drafted following the Standard Protocol for a randomized trial of a social or psychological intervention (CONSORT-SPI). Figure 1 is a general flow chart of the procedure (supplementary material 1 includes the SPIRIT checklist).

### Participants: eligibility criteria

The intervention will be directed exclusively to students of the 10^th^ and 11^th^ grades who are enrolled in one of the education establishments that depend on the Municipality of Santiago. The inclusion criteria for these schools will be the following: (i) have students of these grades enrolled; (ii) not being an education center oriented to adults or persons deprived of liberty (e.g., closed or semi-closed compounds of Gendarmería de Chile or the National Service for Minors); (iii) have regularity in teaching and learning (for example, schools that were not partially interrupted in 2022 by student manifestations); and (iv) have informed consent of the director. The inclusion criteria for the students will be (i) having 90% or greater attendance in classes: (ii) providing informed consent of voluntary participation; and (iii) having explicit and informed consent of their parents or guardians.

The selected students will be randomly divided into the study and control groups; the intervention will take place in the same schools that the students attend. The assignment to groups will use simple random sampling, performed in two stages. In the first stage, the available schools will be randomly assigned to the study and control groups, while the second stage will randomly select the groups in each school that will participate. The study groups will receive a 10-session intervention in sexual education, covering topics related to preventive sexual conduct, gender equity, and mental health. The control groups will not receive intervention; they will continue with the sexual orientation that each school provides to its students. Sexual orientation is general and is provided by the Ministry of Education. The goal is to evaluate and diagnose the behaviors and reproductive health needs of adolescents, addressing the values adopted regarding the decision to engage in sexual relationships and/or have multiple sexual partners, as well as perceptions of risk.

### Study setting

Interventions will be performed in 10 schools financed by the State located in the commune of Santiago (Metropolitan Region, central Chile), which is the third-most populous commune according to the latest country census.

### Sample size

There are currently 6518 students enrolled in the 10^th^ and 11^th^ grades in State-financed schools in the commune of Santiago. Using a 99% confidence level and a 5% error margin, the sample framework is 605 students, which will be divided into the study and control groups. The statistical power is determined using an analysis of variance with a significance level of 0.05, a sample size of 605 and an expected difference of 0.2 points between groups, which will provide a statistical power of at least 0.8. The 0.2 difference is the smallest reported in previous studies comparing the scores of two groups with the instruments that will be used; it was reported in the analysis of the scores of the scale of ambivalent sexism [24].

The statistical power was calculated using the software G*Power [25]. After making a slight adjustment due to the number of students currently enrolled in each school at each level, the definitive size of the experimental phase of the study will be 609 students.

### Randomization

The assignment of the participants to the groups will be implemented in a two-stage selection of the students; since there are already existing teaching-learning processes in process in the schools, with their time limitations, we will have to choose entire “courses” (in Chilean secondary schools, a course is a group of about 40 students who have all classes together) rather than choose individual students.

We will establish groups among the schools to compensate for their different sizes; some schools in the commune are much larger than others (four or more courses per grade. Thus, we will divide the schools into two blocks: one with the largest schools and one with the medium- and small-sized schools. In the second stage, courses that meet the inclusion criteria to participate in the study will be selected randomly within each block. Table 1 details the distribution of the samples.

**Table 1:** Samples to be selected

	**Study group** **No. of students per grade**		**Control group** **No. of students per grade**		
**School**	*10th grade*	*11th grade*	*10th grade*	*11th grade*	Total
*1*	---	---	31	28	59
*2*	---	---	33	27	60
*3*	---	---	34	34	68
*4*	---	---	26	30	56
*5*	---	---	33	32	65
*6*	29	27	---	---	56
*7*	29	30	---	---	59
*8*	28	29	---	---	57
*9*	25	31	---	---	56
*10*	*40*	*33*	---	---	73
	151	150	157	151	
**Sample totals**	**301**		**308**		**609**

### Allocation process

The allocation sequence will be generated using random numbers produced by the statistical program R. A single investigator will carry out both the sequence of participants’ assignments and their assignment to each group (A.A-B.).

To ensure a blind analysis, the professional in charge of data analysis (A. A-B.) will not know to which group the participants belong. Therefore, the database will be anonymized by assigning codes, which will only be known by another team professional (L.L.). Unmasking is not considered to be permitted under any circumstances.

### Awareness of assignment

Before starting the study, collaboration agreements will be signed with the institutions interested in receiving the designed intervention. Subsequently, after identifying the study sites, their consent to participate in the study will be obtained.

Once the schools, courses, and students who will participate in the study have been defined, the school principals, teachers, and students will be contacted to explain the objectives of the intervention, the way it will be performed, and the measurements that will be taken. Since the evaluation instruments will be answered online, the students will be contacted by telephone or WhatsApp to ensure both their participation and follow-up. The final follow-up of the cohort is tentatively scheduled for August 2024.

Since the GRTs is a mixed experimental design, the next phase will evaluate the implementation of the program, in order to analyze how the implementation of a comprehensive, preventive program of sexual health facilitates or impedes a gender-transformative intervention. For this we will conduct semi-structured interviews with those persons who will perform the intervention (teachers, psychologists or guidance counselors) and focus groups of students who participate in the intervention.

These techniques will allow collating and triangulating the results and exploring the particulars of each case, which will complement the findings of the GRTs. The interviews and focus groups will approach the relations between different dimensions of the implementation process and the results experienced by the participants from the gender-transforming approach (benefits for adolescent females, males, and LGTB students, changes in gender norms and stereotypes, among others).

Finally, since this is a mixed study, the information from the experimental phase and implementation will be integrated. This implies analyzing the qualitative results of the trial and then determining how the qualitative results empower the trial [23]. Thus the results obtained from the GRTs may be explained using the information from the experiences of the participants about the implementation process of the program.

### Intervention: description of Focus-on

Focus-on is a comprehensive program of sexual education for adolescents. Its design follows the guidelines of the Intervention Mapping Approach [26] and the orientations proposed by the Framework for Gender-Transformative Health Promotion [16]. Specifically considered are (i) the theories, contents, and strategies of the randomized controlled trial of comprehensive interventions for adolescents performed in the last 10 years [27]; and (ii) the results of a previous study in the object population on the implementation of an intervention in sexual health education. Both studies were done by the team that created the program.

The program is based on three fundamental theories as transverse axes: the Theory of Planned Behavior [28], the Framework for Gender-Transformative Health Promotion [16], and the Ecological Focus of Human Development [29]. Some sessions will use the precepts of the Theory of Social Learning [30], the Cognitive-Behavioral Theory using the emotion-regulating model of Gross [31], and the Transtheoretical Change Model [32].

The intervention has a participative focus, thus the activities are designed to promote processes of knowledge, understanding, application, analysis, and reflection in the adolescents. The activities aim to improve the capacity for critical thinking, favor the retention and communication of the new information, increase the motivation to listen and learn actively, and to improve interpersonal abilities. The execution of the intervention uses three pillars: (i) preventive sexual behavior; (ii) gender equity; and (iii) mental health. These pillars approach their different objectives -promote preventive sexual behavior, gender equity in sexual health, and improve aspects of mental health related to sexuality.

The intervention consists of ten weekly sessions, each lasting 1 h, which (except the first, which is an introduction to the program) treat these pillars gradually, from the basic to the most advanced aspects. The teams that will perform the intervention will be composed of at least two professionals of the psychosocial area (mainly psychologists, social workers, and/or guidance counselors) of the schools included in the study, who will be capacitated in the correct implementation of Focus-on.

Based on the nature of this trial, no relevant concomitant care is required. There is also no specific treatment allowed or prohibited during the trial. Similarly, no particular retention strategy is foreseen, and no special criteria exist for interrupting or modifying the assigned interventions.

### Outcomes

Before implementing the workshop, we will perform a base-level measurement of the variables in the students. Later we will measure the result variables in two moments, just after the workshop closes and 3 months after the closure, in order to evaluate the results obtained by the intervention, to determine the effect of the program.

The result variables of the intervention will be the following: (a) change in knowledge of sexuality manage correctly information on topics related to the biological components of sexuality such as reproduction, prevention of STI and pregnancy, among others; (b) change in preventive sexual behavior, which may be related to (i) behavior or intention to prevent STI and pregnancy, or (ii) sexual abstinence or intention to postpone initiation of sexual life; (c) change in gender attitude related to (i) positive disposition towards persons of different genders and sexual orientation, and (ii) empowering in sexuality of LGBT and female students; (d) change in components of mental health related to sexual health, decrease of internalizing-externalizing symptomology, and improvement in self-efficacy (Table 2). The following instruments will be used:

- *Knowledge test* [33]. This is a 34-item multiple choice instrument which was designed to evaluate the effectiveness of interventions in sexual education to improve knowledge on sexuality in adolescents; it evaluates areas including pregnancy, STI, physical development, and others. Its reliability is *α* = 0.89.
- *Ad hoc survey*. It was elaborated by the research team to evaluate the behavior and intention of preventive behavior in sexuality, self-efficacy. and empowerment in sexuality in LGTB and female students. Ad-hoc surveys have been used and reported in other randomized controlled trial of interventions in sexual health (e.g.. Coyle [34], Goesling [35]. and Yakubu [36]). This survey will follow the guidelines developed by Manaseri [37] and Jirapongsuwan [38].
- *Scale for detection of sexism in adolescents (DSA)* [39]. This is an instrument of 26 items developed for adolescents. It evaluates sexism (attitude of superiority towards women), differentiating between hostile and benevolent sexism. The response scale for the items is a Likert type with six alternatives (from 1 = disagree completely to 6 = agree completely). Its reliability is *α* = 0.881.
- *Scale of negative attitudes toward trans persons (NATP)* [40]. It measures attitude as an expression of prejudice towards trans persons. It has nine items with a Likert scale with five response options, from 1 = disagree completely to 5 = agree completely. Its reliability is *α* = 0.886.
- *Short version of modern homophobia scale* [41]. It measures homophobic attitudes in the dimensions of personal discomfort, institutional, and deviation/changeability. It is composed of 46 items that evaluate attitudes towards lesbians and gays on a Likert scale of 1–5. Its reliability is *α* = 0.80.
- *Pediatric symptom checklist (PSC-17)* [42]. This is an abbreviated version of the 35-item PSC [43]. It evaluates general psychosocial functioning, detecting emotional and behavioral difficulties in children and adolescents [44]. Its reliability is *α* = 0.72. PSC-17 is self-administered by adolescents, evaluating the existence of internalizing difficulties (e.g., despair and sadness), externalizing difficulties (e.g., difficulties in social relations such as disobedience of norms and fights with others), and attention difficulties (e.g., difficulty in concentration -60,66-). As in other studies (e.g., Penner [45]), this instrument will be used to evaluate the adolescents’ mental health. The baseline and follow-up evaluations will be obtained confidentially, and be recorded in files managed by the team responsible for the study.

**Table 2:** Enrollment, intervention, and assessments

	**Instruments**		**Baseline**	**Post**	**3 months**
**Timepoint**		−*t*_1_	*t*_1_	*t*_2_	*t*_3_
**Enrollment**					
Informed consent		x			
Assent		x			
Allocation		x			
**Intervention**					
School-based sexual education intervention					
**Assessments**					
Socio-demographic	Personal and home information (ad hoc survey)		x		
	Social network information		X		
Knowledge about sexuality; preventive behavior in sexuality and behavioral intention; empowering and self-efficacy	Knowledge test [46, 47] Survey ad hoc		x	x	x
Gender attitude	Scale of detection of sexism in adolescents (DSA) [48]	x	x	x	x
	Scale of negative attitudes towards trans persons [40]	x	x	x	x
	Short version of Modern Homophobia Scale [41]	x	x	x	x
Mental health	PSC-17 [42]	x	x	x	x

The enrollment, intervention, and assessments are shown in Table 2.

In each intervention session, we will record the attendance and duration, and using structured observation [49], we will record some elements referring to the implementation of the intervention. Those responsible for executing the intervention will receive bi-monthly supervision to control the evaluation and retention.

No specific retention strategy is planned. Available information for students who drop out will include their socio-demographic data, the number of sessions attended, and the results obtained in the tests administered before dropping out.

Because it will be a mixed experimental design GRTs, at the end of the intervention, we will perform a qualitative Case Study to ascertain the experience of the participants and executors with respect to the program and its implementation. Each school of the study group will be a case. The participants will be students who participated in the program and the executors who implemented the intervention. Each case will include focus groups with the students (at least one per case) and semi-structured interviews with the executors (at least two per case). We will then perform a Qualitative Content Analysis to identify certain emergent topics.

### Analytical methods

The information that results from the application of the scales will be summarized with descriptive statistics (frequency table, mean, standard error, and asymmetry coefficient) for the pre- and post-intervention evaluations.

Since the intervention has specific objectives for each session, it is not appropriate to impute missing data. However, to account for the potential lack of adherence by some students, it is suggested that participants who miss more than 30% of the sessions should not be included in the data analysis. Regardless of attendance, students will be able to participate in all workshop activities.

We will compare the three evaluations, incorporating information relative to the type of school and course. If the data are normally distributed, we will perform one-way ANOVAs, incorporating post hoc information of the results when necessary, and perform pair-wise comparisons using the Tukey or Games-Howell test.

After the variables with main effects are established, we will perform a factorial ANOVA, in which one of the factors will be fixed (i.e., type of school) and the other will be the repeated measures of the three evaluations. As well as evaluating if the main effects are sustained over time, this will evaluate possible interactions among the studied factors. In this case, following the guidelines proposed by Cohen^75^ for ANOVAs with two or more factors, the effect size will be reported as the partial Eta^2^ statistic. The interpretation of the effect sizes will consider the suggestions of Cohen [50]. These analyses will use the program Jamovi version 1.2 [51].

No interim data analysis will be conducted, and there will be no stopping guidelines.

### Ethics

This study implies a minimum risk for the adolescents, with the possibility of a direct benefit, since the probability and magnitude of damage and the discomfort possible to be foreseen in the research are no greater than those found commonly in the daily life or during physical or routine psychological tests [46]. We will establish confidentially in the application of the instruments and the presentation of the results. This will be recorded and signed both in the informed consent of the adolescents and in that of the parents or legally responsible adults. Participants will have the option of withdrawing from the study at any moment; they will be informed of its purpose and the way the results will be used. The authors will meet every 2 weeks to supervise the application of the study and the clinical and research integrity. If modifications need to be made to the protocol, the study committee will notify the Municipality of Santiago, the participants, and update the trial registry.

Results of the trial will be submitted for publication in peer-reviewed journals and findings will be presented at scientific conferences and to key service providers and policymakers. The study was approved by the Ethics Committee of the Faculty of Social Sciences of the Universidad de Chile, the institution to which the principal and corresponding researcher belongs. The committee will monitor and safeguard the data.

## Discussion

Sexual and reproductive health are relational processes of construction in which the social context and individual, family, and community behavior intervene. Because of this, world organizations recommend an integrated focus of promotion of sexual health, with effective participation of all sectors of the society and the combination of complementary multi-sector strategies, to ensure informed and healthy populations by the application of interventions that promote safe sexual behavior [47]. This is especially sensitive in adolescence, since this stage is the opportune moment to develop healthy habits and lifestyles related to sexual health.

This stage is a period of emotional and social change, in which adolescents begin to explore sexuality and relate actively to others. In fact, an important proportion is sexually active, and this proportion increases in late adolescence [3]. Thus education in sexual and reproductive health during this stage of the life cycle is key, since the information and knowledge are central to self-care and the incorporation of preventive measures in the exercise of sexuality [47]. Thus initiatives are required to evaluate the design and implementation of interventions and/or programs that contribute to improve the health of this population in this ambit.

This study presents the study protocol of an GRTs study of an intervention in the sexual health of Chilean adolescents, centered mainly on evaluating the effectiveness of an intervention with a comprehensive and gender-transforming perspective. Given the responsibility of public health in adolescent sexual and reproductive health, the provision of resources and options to educate in an integral, scientific manner with a contextualized gender focus becomes an imperious need.

The development of interventions on this topic is urgent, given the magnitude of non-intended adolescent pregnancy, the high rates of STI in the adolescent and young adult population (which represent 55% of the cases notified in the last 10 years) and the progressive growth in the HIV notification rate in adolescents. The prevalence of risky sexual behavior shows that the increase in self-care measures has been unequal in men and women [47]. Adolescents have reported that the information they receive from schools, health professionals, and their families is insufficient, thus they turn to friends, television, and the Internet, in spite of knowing the limitations of these sources [52].

Thus interventions are required that offer friendly, quality spaces for adolescents, with emphasis on sexual and reproductive health, ensuring sexual education in the education structure and incorporating the family and community as socializing and protective agents [47]. Women, men, and persons of sexual diversity should have the same opportunities to accede to life conditions and services to maintain good health, without becoming ill, incapacitated, or dying for avoidable causes [47].

The innovative aspects of the proposal should be highlighted. This study is proposed based on Implementation Science, a relatively recent framework of study on the processes to be put in practice to make effective an evidence-based intervention in a new context [53], whose results will serve as necessary preconditions to produce changes, since they will indicate what is or is not successful in the execution of an intervention [54]. The proposal also contributes to fill a gap in this aspect, since there are few public reports on the implementation of interventions in schools, particularly in sexual health [55].

Another innovative aspect is the use of Gender-Based Analysis, a recent practice in health research [56] and in implementation studies [48]. This kind of analysis implies an analytical process that examines how gender impacts the effectiveness of the interventions, considering social norms and economic and cultural conditions and evaluating how and why different subpopulations (e.g., groups considering their gender, age or socioeconomic status) experiment certain conditions of health and interventions [21].

## Trial status

This study was registered on June 6, 2023, at ClinicalTrials.gov ID: NCT05896540. Recruitment will begin in September 2023 and end in July 2024. Protocol version number 1.0. May 22, 2023.

### Supplementary Information

Supplementary Material 1.

## Data Availability

The data that will be generated and analyzed during the current study will be available from the corresponding author upon reasonable request.
